# Necroptosis throws novel insights on patient classification and treatment strategies for hepatocellular carcinoma

**DOI:** 10.3389/fimmu.2022.970117

**Published:** 2022-07-27

**Authors:** Bowen Hu, Jie Gao, Jihua Shi, Feng Zhang, Chengcheng Shi, Peihao Wen, Zhihui Wang, Wenzhi Guo, Shuijun Zhang

**Affiliations:** ^1^Department of Hepatobiliary and Pancreatic Surgery, The First Affiliated Hospital of Zhengzhou University, Zhengzhou, China; ^2^The First Affiliated Hospital of Zhengzhou University, Henan Research Centre for Organ Transplantation, Zhengzhou, China; ^3^The First Affiliated Hospital of Zhengzhou University, Henan Diagnosis & Treatment League for Hepatopathy, Zhengzhou, China; ^4^The First Affiliated Hospital of Zhengzhou University, Henan Engineering & Research Center for Diagnosis and Treatment of Hepatobiliary and Pancreatic Surgical Diseases, Zhengzhou, China; ^5^Department of Pharmacy, The First Affiliated Hospital of Zhengzhou University, Zhengzhou, China

**Keywords:** necroptosis, hepatocellular carcinoma, multi-omics, immunity, drugs

## Abstract

**Introduction:**

Necroptosis is a novel pattern of immunogenic cell death and has triggered an emerging wave in antitumor therapy. More evidence has suggested the potential associations between necroptosis and intra-tumoral heterogeneity. Currently, the underlying role of necroptosis remains elusive in hepatocellular carcinoma (HCC) at antitumor immunity and inter-tumoral heterogeneity.

**Methods:**

This study enrolled a total of 728 HCC patients and 139 immunotherapy patients from eight public datasets. The consensus clustering approach was employed to depict tumor heterogeneity of cancer necroptosis. Subsequently, our study further decoded the heterogeneous clinical outcomes, genomic landscape, biological behaviors, and immune characteristics in necroptosis subtypes. For each patient, providing curative clinical recommendations and developing potential therapeutic drugs were used to promote precise medicine.

**Results:**

With the use of the weighted gene coexpression network analysis (WGCNA) algorithm, necroptosis-associated long non-coding RNAs (lncRNAs) (NALRs) were identified in HCC. Based on the NALR expression, two heterogeneous subtypes were decoded with distinct clinical outcomes. Compared to patients in C1, patients in C2 harbored superior pathological stage and presented more unfavorable overall survival and recurrence-free survival. Then, the robustness and reproducibility of necroptosis subtypes were further validated *via* the nearest template prediction (NTP) approach and classical immune phenotypes. Through comprehensive explorations, C1 was characterized by enriched immune-inflammatory and abundant immune infiltration, while C2 possessed elevated proliferative and metabolic activities and highly genomic instability. Moreover, our results indicated that C1 was more prone to obtain desirable benefits from immunotherapy. For patients in C2, numerous underlying therapeutic agents were developed, which might produce significant efficacy.

**Conclusion:**

This study identified two necroptosis subtypes with distinct characteristics, decoding the tumor heterogeneity. For an individualized patient, our work tailored corresponding treatment strategies to improve clinical management.

## Introduction

Hepatocellular carcinoma (HCC) is a common global cancer burden and is the third most prevalent cause of cancer-related mortality worldwide, accounting for over 700,000 fatalities per year (1). Owing to its advanced malignant and high aggressive characteristics, patients display dismal prognosis, and the 5-year survival rate is only 18% (2). Over the last decade, encouraging advances in cancer treatments have been achieved, such as surgical resection, immunotherapy, targeted treatment, and chemoradiotherapy (3). In practice, various treatment choices are provided for HCC patients, but not all approaches consistently have curative efficacy, which can be mainly attributed to tumor heterogeneity. Previous research had suggested that tumor heterogeneity is usually mirrored by distinct molecular characteristics, and more studies have focused on decoding disease heterogeneity to improve clinical outcomes (4, 5). Immunotherapy as an emerging approach has conspicuous benefits by acting on specific molecules, such as *PD1*, *PD-L1*, and *CTLA-4*, while only a subset of subpopulations present desirable efficacy. In addition, the administration of chemotherapy is also unsatisfactory in clinical utility, due to high therapeutic expense and varying degrees of drug susceptibility (6). Enormous evidence had underlined the significant links between tumor heterogeneity and therapy efficacy. Nonetheless, current investigations demonstrate that the exploration of tumor heterogeneity and the description of molecular features remain insufficient. It is imperative to decode tumor heterogeneity and identify distinct characteristics for each patient, facilitating prognosis and therapeutic efficacy.

In recent years, necroptosis as a potentially novel way of immunogenic cell death has gradually become an emerging wave in antitumor therapy (7). In-depth studies have elucidated that necroptosis harbors unique molecular features and is categorized into non-apoptotic cell deaths, which are mainly mediated by *PRK1*, *RIPK3*, *RIP1*, *MLKL*, etc. (7, 8). The role of necroptosis in regulating cancer biology is complicated, encompassing tumorigenesis, cancer metastasis, and antitumor immunity (9). As is well known, tight crosstalk exists between necroptosis and anticancer immunity, and the occurrence of necroptosis could elicit intense adaptive immune responses and amplify antitumor immunity (7, 9). The induction of necroptosis combined with immune checkpoint (ICP) inhibitors (ICIs) displays synergistically enhanced antitumor ability (10). Systematically and comprehensively exploring the relationships between necroptosis and immune tumor microenvironment (TME) is more prone to improving desirable immunotherapy efficacy. In addition, tolerance to apoptosis is linked with drug resistance (11), while necroptosis reportedly serves as a coalescence of necrosis and apoptosis, which contributes to overcoming chemotherapy failure. Therefore, necroptosis might be an underlying target for cancer therapy, and developing drugs to defend against cancer may obtain more clinical benefits based on inducing or manipulating necroptosis.

The long non-coding RNAs (lncRNAs) usually possess over 200 nucleotides that are not translated into proteins. Notably, a growing arsenal of evidence has elaborated that lncRNAs are closely implicated in inflammatory responses, immune infiltration, and immunotherapy (12, 13). Indeed, lncRNAs also regulate gene expression at various levels and are closely associated with programmed cell death, such as necroptosis (14). The depletion of Linc00176 has been elucidated to disrupt the cell cycle and trigger necroptosis by releasing tumor suppressor miRNAs (15). Currently, the role of lncRNA and necroptosis in the antitumor effect remains largely unexplored; integrated analysis might open new insights and throw light on the clinical management of HCC patients.

In our study, the pronounced implications of necroptosis were decoded by exploring its links with the TME. According to gene expression, necroptosis-associated lncRNAs (NALRs) were identified, and further two heterogeneous subtypes were proposed. Subsequently, using three independent databases, the robustness of necroptosis subtypes was rigorously verified *via* the nearest template prediction (NTP) algorithm. Moreover, profound heterogeneities were depicted and uncovered between necroptosis subtypes, including distinct prognosis, biological functions, clinical features, genomic variations, and immune microenvironment characteristics. For each patient, we provided immunotherapy evaluation and developed potential therapeutic drugs, aiming to seek optimal clinical decisions. Overall, this work explored the tumor heterogeneity from NALR perspectives and tailored therapeutic strategy for each HCC patient, which was helpful to improve prognosis and facilitate clinical management.

## Methods

### Data acquisition and processing

In the present study, a total of 728 HCC patients were retrieved from four public databases, including TCGA-LIHC, GSE14520, GSE116174, and GSE10141. The patients were extracted in this study based on the following criteria: a) primary HCC, b) the number of patients in each dataset is over 50, c) not under any preoperative radiotherapy or chemotherapy, and d) complete gene expression profiles and corresponding survival information. In addition, the somatic mutation data were obtained from The Cancer Genome Atlas (TCGA) Genomic Data Commons (GDC) portal, and copy number variation (CNV) data were downloaded from the FireBrowse online tool, which was processed using the genomic identification of significant targets in the Cancer 2.0 (GISTIC2.0) algorithm. Four eligible databases with expression data and immunotherapeutic information were also screened, encompassing GSE35640, GSE91061, GSE100797, and Nathnaon cohorts. According to the Response Evaluation Criteria in Solid Tumors (RECIST) v1.1 standard (16), a total of 98 non-responders and 41 responders were used to assess immunotherapy efficacy. The detailed criteria were as follows: patients who had a complete response (CR) or partial response (PR) and patients who had stable disease (SD) or progressive disease (PD) were regarded as responders and non-responders, respectively, and not evaluable (NE) patients were excluded from our study. For the RNA-seq data, all expression data were converted into transcripts per kilobase million (TPM) and further log-2 transformed. In parallel, the expression data from microarrays were normalized using the robust multiarray average (RMA) approach. The baseline characteristics of all patients were available in **Table S1**.

### The significance of necroptosis in tumor microenvironment

Based on previous literature (17, 18), 115 necroptosis-associated genes were retrieved in our study (**Table S2**). The principal component analysis (PCA) was applied using the prcomp function, and the results were visualized by *scatterplot3d* package. Then, the single-sample gene set enrichment analysis (ssGSEA) algorithm was utilized to estimate necroptosis score and immune infiltration abundance. With the use of the *estimate* package, stromal and immune scores were measured to further evaluate the TME. The correlation was depicted by Spearman’s between necroptosis score and 28 immune cell infiltration, stromal score, and immune score. To explore the underlying links between necroptosis score and immune checkpoint expression, the radar map delineated by the *radarchart* package further exhibited the correlations.

### Identification of necroptosis-associated long non-coding RNAs

The weighted gene coexpression network analysis (WGCNA) was usually used to explore and identify the coexpression gene modules (19). We employed the *WGCNA* package to generate coexpression necroptosis-associated lncRNA networks of TCGA-LIHC. After the outlier samples were excluded, the expression matrix of the top 5,000 genes was converted into an adjacency matrix, and further unsupervised coexpression relationships were constructed. Based on the scale-free topology criterion, an appropriate power β (soft threshold) was calculated to develop a scale-free network. Subsequently, the weighted adjacency matrix was transformed into the topological overlap matrix (TOM) describing the overlap of network neighbors, and the corresponding dissimilarity 1 − TOM was produced. Then, gene modules from the system cluster tree were identified by a dynamic tree cutting approach. To recognize lncRNA modules prominently associated with necroptosis score, the module with the highest correlation was filtered for subsequent analysis.

### Development of molecular subtypes

With the use of univariate Cox regression, the modules that contained necroptosis-associated lncRNA genes, which harbored the most significant correlation, were further filtered to generate prognosis-associated candidate genes. According to the expression profiles of these genes, consensus clustering was employed to develop clusters in TCGA-LIHC dataset. This process was performed by the Kmeans method implemented in the *ConsensusClusterPlus* package (20). The detailed parameter setting has the following criteria: a) subsample of 80% of samples at each iteration, b) possible cluster ranks = 2–9, c) the number of iterations = 1,000, and d) Euclidean distance. To determine the optimal number of molecular clusters, the consensus score matrix, proportion of ambiguous clustering (PAC) score, and cumulative distribution function (CDF) curve were synthetically executed for explorations. Subsequently, the silhouette coefficient was employed to quantify the robustness of clustering patterns, and a higher silhouette value means a better match to its own pattern (21).

### Nearest template prediction approach verifies the distinct subtypes

For necroptosis subtypes, the prognosis value was assessed by survival curves of overall survival (OS) and recurrence-free survival (RFS). The molecular characteristics were also deciphered by gene set variation analysis (GSVA), which was broadly applied in pathway activities exploration (22). With the use of 50 Hallmark gene sets from Molecular Signatures Database (MSigDB), distinct biological characteristics were elucidated in necroptosis subtypes. In addition, a classical six immune subtypes had been proposed across 33 diverse cancer types from the perspective of extensive immunogenomic analysis (23). The *ggSankeyGrad* and *survival* packages were implemented to depict the underlying links between immune clusters and necroptosis subtypes.

The NTP approach is flexible for assessing class prediction confidence for each patient (24). The signature gene list was obtained from gene modules with high correlation by WGCNA and then utilized in the NTP algorithm implemented in the *CMScaller* package (25), evaluating the reliability and stability of necroptosis subtypes.

### The landscape of genomic variations and clinical characteristics

To further depict the landscape of genomic variations, we decoded molecular heterogeneity at the genomic level. Several previous studies (26, 27) had suggested that frequently mutated genes (FMGs) that harbored top 20 mutational frequencies were regarded as major driver genes, such as *TP53* and *CTNNB1*. With the use of the *maftools* package (28), the tumor mutational burden (TMB) of each patient was calculated, and the overview of mutation frequency was displayed. Then, the FMGs were compared between necroptosis subtypes. Based on CNV data from GISTIC2.0 analysis, we also dissected the burden of amplification and deletion at focal and arm levels and quantized the percentage of genetic changes, including the fraction of genome alteration (FGA), fraction of genomic gained (FGG), and fraction of genome lost (FGL). Apart from TMB, aneuploidy score and homologous recombination deficiency (HRD) were also compared in necroptosis subtypes, further identifying latent genomic features. In addition, clinical characteristics were also explored in necroptosis subtypes, including age, gender, and American Joint Committee on Cancer (AJCC) stage. The multivariate Cox regression was implemented to determine independent prognostic indicators for OS and RFS.

### Delineate the immune landscape and assessment of immunotherapy

Gene expression profiles were further exploited to decode the TME characteristics of each HCC patient. To depict a more detailed landscape of immunological TME, we used the ssGSEA algorithm to estimate the relative infiltration abundance of 28 immune cell subgroups. The expression of ICPs was used to evaluate the immune state of a single sample, which contained 27 molecules from the B7-CD28 superfamily, TNF superfamily, and other molecules. The co-stimulatory and co-inhibitory ICPs were also applied to explore the differences in necroptosis subtypes. The HLA molecules and leukocyte fraction were employed to decipher the capacity of antigen presentation and degree of inflammatory infiltration, respectively. In parallel, T-cell inflammatory signature (TIS) with 18 immune genes was measured *via* the ssGSEA algorithm, and unsupervised subclass mapping (Submap) was used to appraise the expression profile similarity, further predicting immunotherapeutic efficacy in distinct necroptosis subtypes. Four immunotherapy datasets were executed to reveal clinical implications underlying distinct subtypes, and the robustness of therapy evaluation was examined using the receiver operating characteristic (ROC) curve.

### Potential therapeutic drugs

To develop underlying therapeutic drugs, the half-maximal inhibitory concentration (IC50) of each patient was calculated by the *pRRophetic* package, which is popularly used in predicting drug response. A lower IC50 value means a higher drug sensitivity. Thus, potential therapeutic agents were identified when patients possessed a lower IC50 value among necroptosis subtypes. Moreover, the Connectivity Map (CMap) is also a wide-utility approach for searching for potential therapeutic drugs and targeted pathways according to gene expression profile similarity. The elevated expression genes of specific necroptosis subtypes were screened *via* the *limma* package, and the expression similarity with database signatures was compared. Then, the enrichment score was quantified to assess the therapeutic sensitivity and provide potential therapeutic drugs.

### Statistical analysis

All data cleaning, statistical analysis, and visualization were conducted in R 4.1.2 software. Spearman’s correlation analysis was utilized to elucidate the relationships between two continuous variables. The Kaplan–Meier approach and Log-rank test were executed to measure the different OS and RFS between the two groups. The t-test or Wilcoxon rank-sum test was adopted to evaluate the differences when comparing two continuous variables. Pearson’s chi-squared test or Fisher’s exact test was exploited to compare categorical variables. With the use of the *survminer*, *survival*, and *pROC* packages, the determination of optimal cutoff values, Cox regression analysis, and ROC curve for predicting binary categorical variables was carried out, respectively. The p < 0.05 was considered statistically significant, and all statistical tests were two-sided.

## Results

### The role of necroptosis in hepatocellular carcinoma

**Figure 1:**
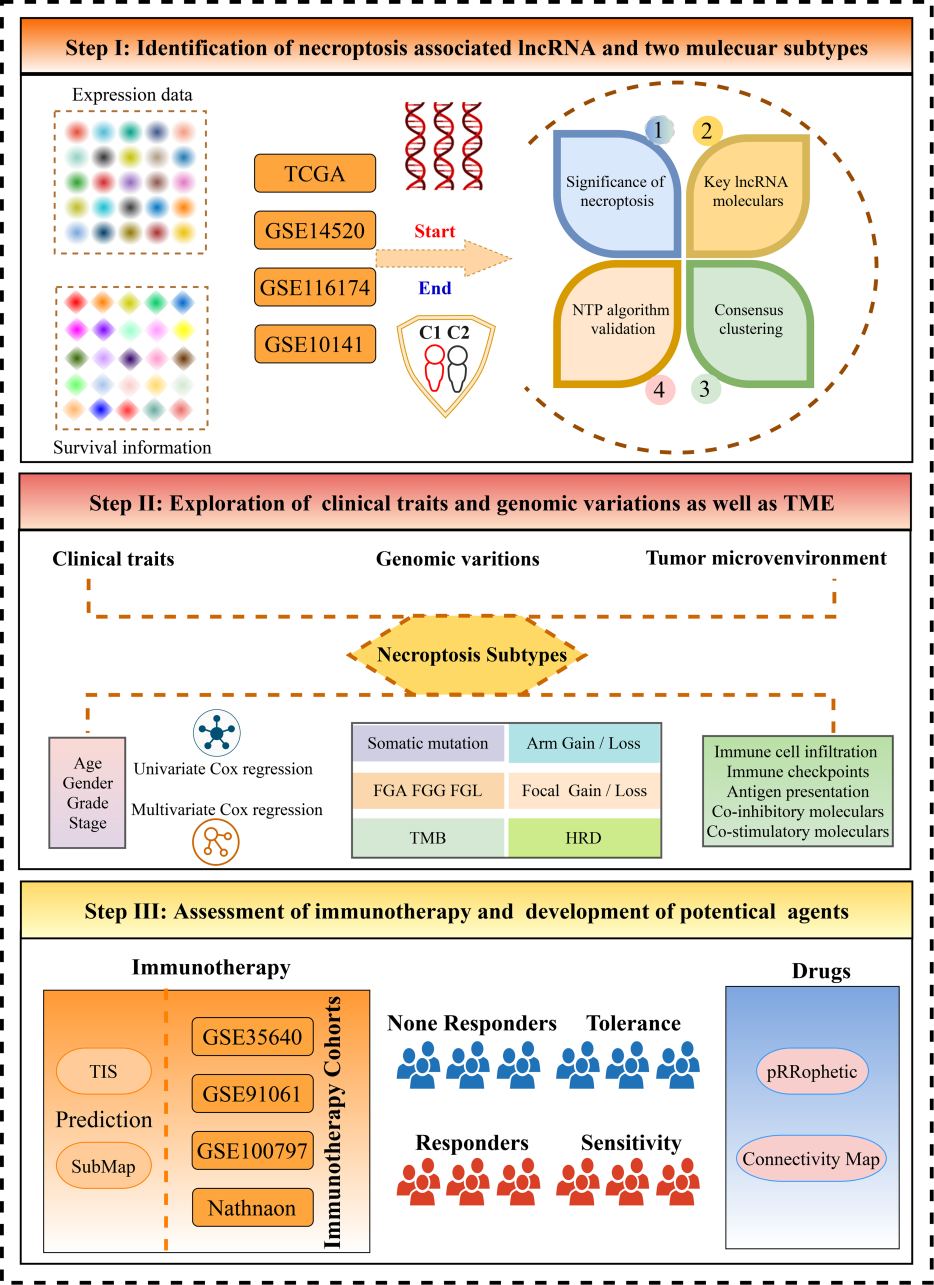
The flow diagram of this study.

**Figure 2:**
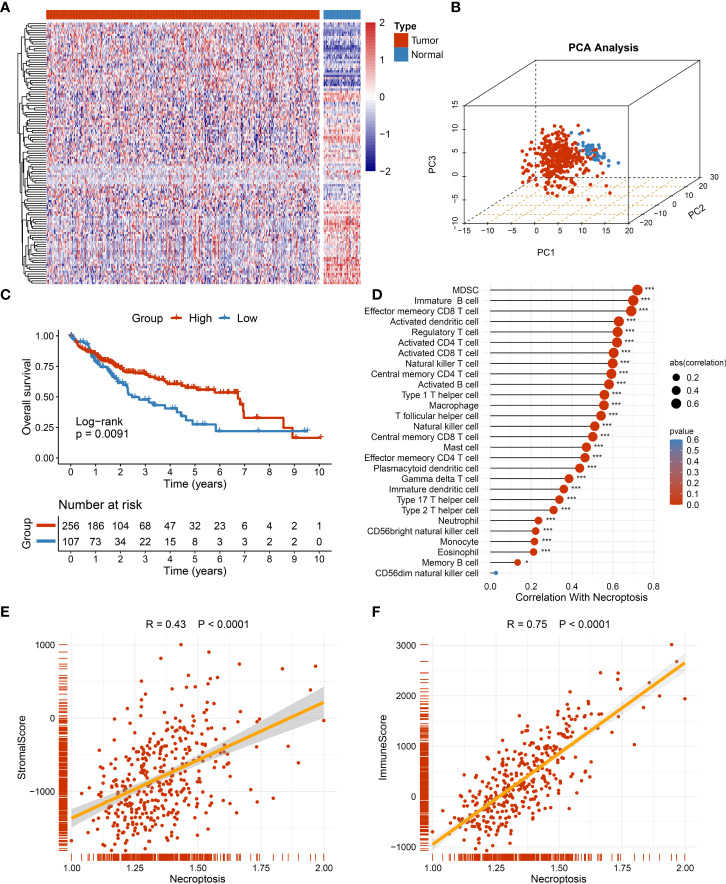
The role of necroptosis in tumor microenvironment (TME). **(A)** The expression of 115 necroptosis-associated genes between tumor and normal tissue. **(B)** The distribution of all samples using principal component analysis (PCA) in TCGA-LIHC dataset. **(C)** Kaplan–Meier curves of overall survival (OS) according to the necroptosis score in TCGA-LIHC dataset. **(D)** Correlations between 28 immune cell infiltration and necroptosis score using Spearman’s analysis. **(E)** Correlations between stromal score generated from estimate algorithm and necroptosis score. **(F)** Correlations between immune score generated from estimate algorithm and necroptosis score. *p < 0.05, ***p < 0.001.

**Figure 3:**
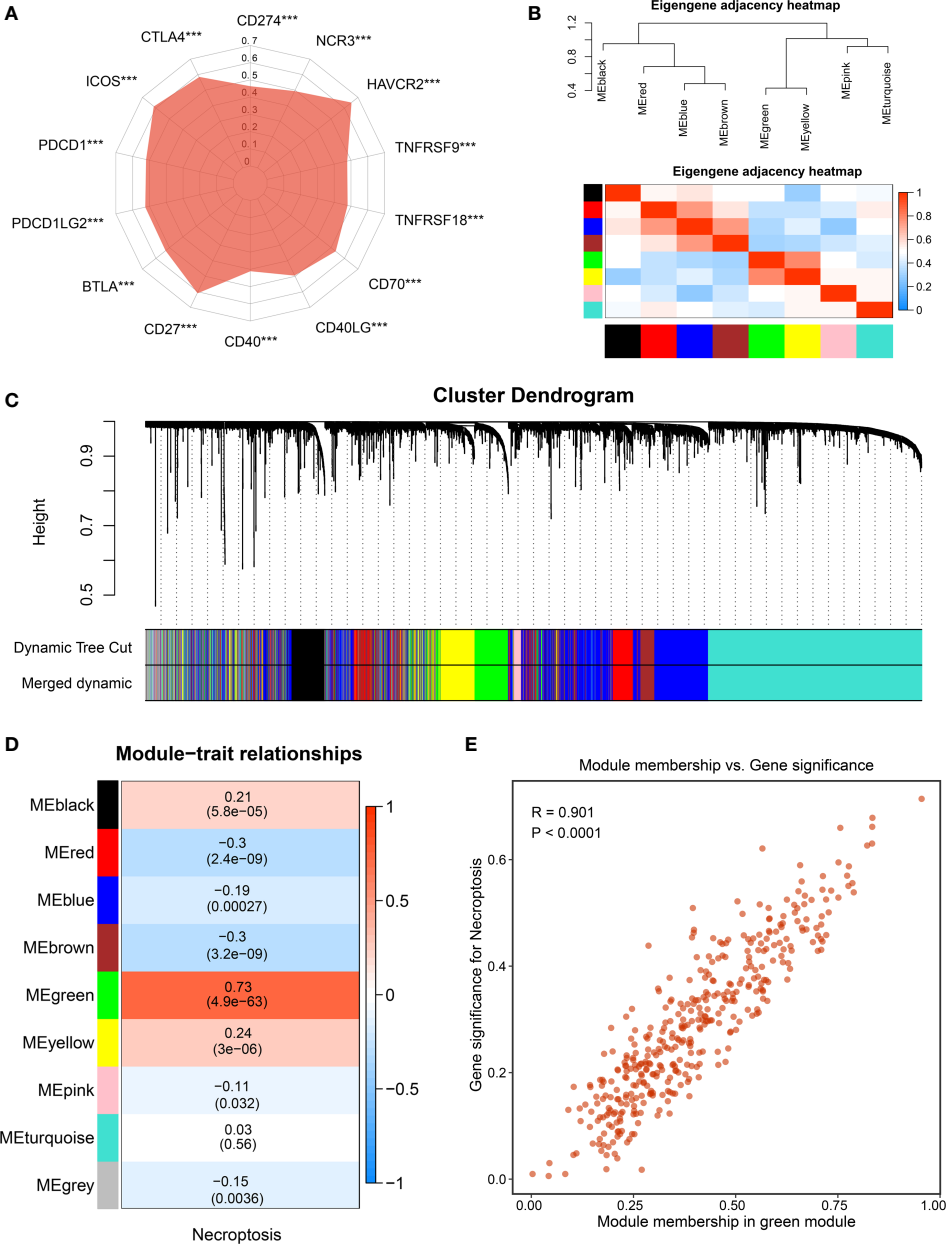
The identification of necroptosis-associated lncRNAs. **(A)** Correlations between the expression of immune checkpoint molecules and necroptosis score. **(B)** The heatmap reveals the eigengene adjacency of distinct modules. **(C)** Clustering dendrograms of co-expression network modules; each module was assigned a color. **(D)** Correlation analysis between gene modules and necroptosis score. **(E)** The scatterplot of module membership (MM) vs gene significance (GS) of the necroptosis score. ***p < 0.001.

The workflow of the current study is shown in **Figure 1**. Through systematic and comprehensive investigations, 115 necroptosis-associated genes (NAGs) were extracted to explore necroptosis. Based on NAG expression, our study discovered significant differences between tumor and normal tissues, hinting at distinct biological behaviors (**Figure 2A**). In TCGA-LIHC dataset, samples from tumor and normal tissues could be well distinguished by NAG expression profiles, indicating that necroptosis caused tumor heterogeneity and might have crucial roles in HCC tumorigenesis (**Figure 2B**). More studies had elucidated that TME harbored tight relationships with tumor initiation and progression. Thus, the necroptosis score was calculated to decode the crosstalk with TME using the ssGSEA algorithm. The higher necroptosis score displayed a more favorable prognosis (**Figure 2C**) and superior immune cell infiltration (**Figure 2D**). In addition, the necroptosis score presented strong correlations with both stromal score and immune score, implying latent power of regulating TME (F**igures 2E, F**). There were also prominently positive correlations with common immune checkpoints, such as *CD274* and *CTLA4* (**Figure 3A**). All the above results suggested that necroptosis possessed intense connections with TME and might have a conspicuous impact on tumorigenesis.

### Screening necroptosis-associated long non-coding RNAs and developing heterogeneous subtypes

To further decode tumor heterogeneity, our study identified NALRs *via* the WGCNA approach. First of all, the sample dendrogram and trait heatmap were visualized, and then outlier samples were removed to conduct clustering (**Figure S1A**). A scale-free network was developed when soft threshold β was set as 4, and the no-scale R^2^ was close to 0.9 (**Figure S1B**). Then, through cluster dendrogram and eigengene adjacency heatmap, cutting and clustering were applied to the samples, resulting in eight coexpression modules (**Figures 3B, C**). Furthermore, the relationships between gene modules and necroptosis scores were measured. Among these, the green module displayed the strongest links with the necroptosis phenotype (**Figure 3D**). The robustness of gene modules was ultimately tested *via* correlation analysis between gene significance (GS) and module membership (MM) (**Figure 3E**). Significant R value indicated intense links, and the green module genes were defined as NALRs for performing follow-up works.

**Figure 4:**
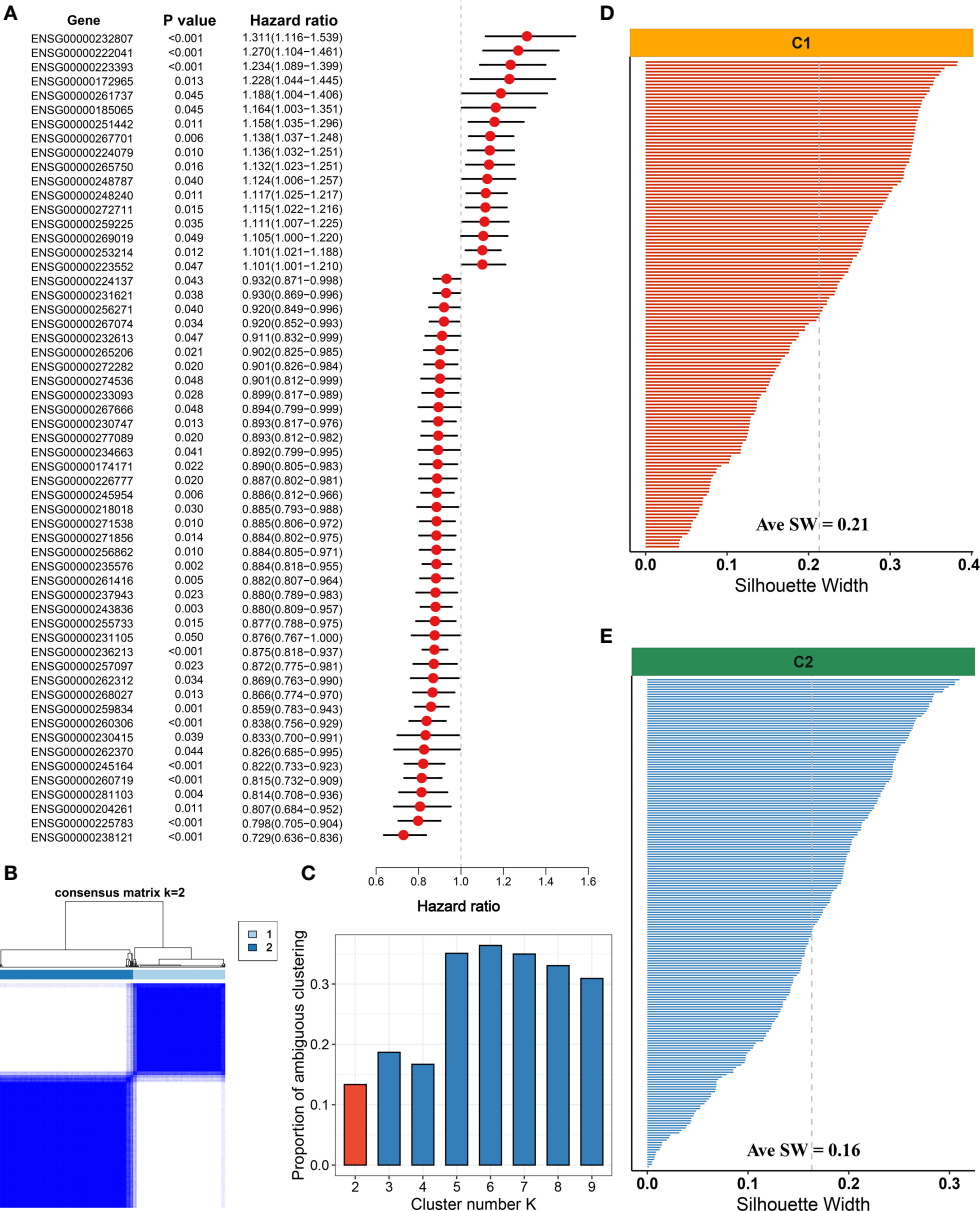
Development of necroptosis subtypes by consensus clustering in hepatocellular carcinoma (HCC). **(A)** Univariate Cox regression analysis of necroptosis-associated lncRNAs (NALRs). **(B)** The consensus score matrix of necroptosis subtypes using consensus unsupervised clustering. **(C)** The first rank (k = 2) in which proportion of ambiguous clustering (PAC) score displays the lowest was generally defined as the optimal rank. **(D)** The silhouette statistic of necroptosis subtype with C1. **(E)** The silhouette statistic of necroptosis subtype with C2.

With the use of univariate Cox regression analysis, a total of 57 prognosis-associated NALRs were screened for exploring HCC heterogeneity (**Figure 4A**). According to the NALR expression, consensus cluster analysis was employed to decipher heterogeneous subtypes, in which all HCC patients were initially assigned to k (k = 2–9) clusters. A higher consensus score means more likely to divide into the same subgroup (**Figure 4B** and **Figure S2B–H**). In parallel, the smoother middle segment of the CDF curve represents clearer sample assignments (**Figure S2A**). All results suggested that the optimal clustering number was generated when k = 2. Moreover, the PAC was also popularly used to assess unsupervised clustering by quantifying the middle segment. When samples were assigned into two clusters, PAC presented the lowest value, implying k = 2 was the best again (**Figure 4C**). To further identify stable and robust subtypes, samples were detected by measuring silhouette coefficient and screening positive silhouette width (**Figures 4D, E**).

### The significant prognosis value of necroptosis subtypes

**Figure 5:**
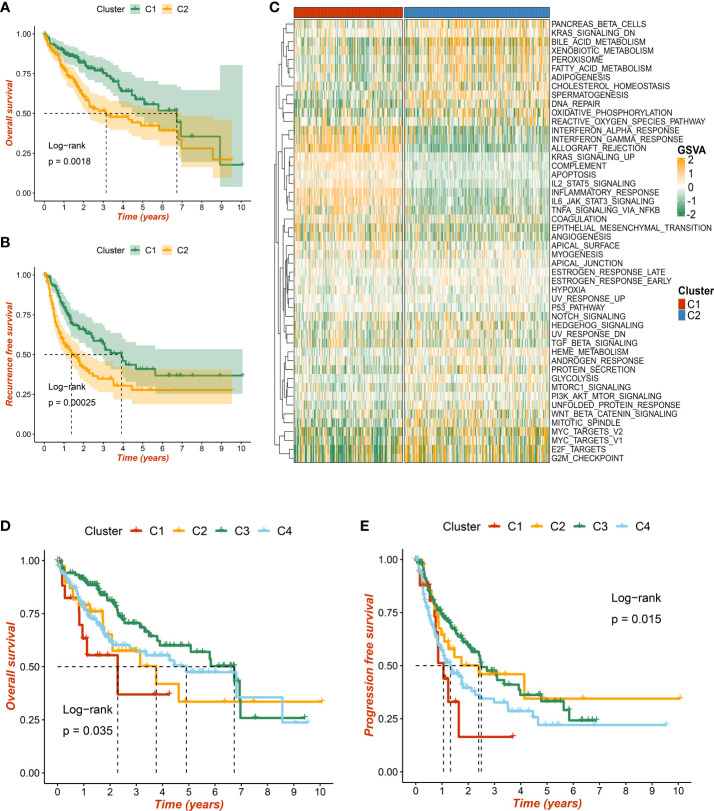
The prognostic significance of necroptosis subtypes. **(A)** Kaplan–Meier curves of overall survival (OS) according to the necroptosis subtypes in TCGA-LIHC dataset. **(B)** Kaplan–Meier curves of recurrence-free survival (RFS) according to the necroptosis subtypes in TCGA-LIHC dataset. **(C)** Heatmap of 50 Hallmark gene sets among three clusters using the gene set variation analysis (GSVA) algorithm. **(D, E)** Based on previous immune subtypes, Kaplan–Meier curves of OS and progression-free survival (PFS) according to different subtypes, respectively.

As mentioned above, our study identified two robust necroptosis subtypes, termed C1 and C2. To enhance clinical utility, the prognosis value of subtypes was further elucidated. The results exhibited that C2 displayed an inferior prognosis at both OS and RFS levels (p < 0.05) (**Figures 5A, B**). Underlying biological characteristics might map onto heterogeneous clinical outcomes; thus, 50 Hallmark pathways were enrolled to decipher the potential biological behaviors of C1 and C2. Interestingly, patients in C1 were mainly enriched in immune-inflammatory pathways, such as interferon-gamma response and inflammation, while patients in C2 were obviously related to the metabolic and proliferative activities (**Figure 5C**). Therefore, C1 was characterized as immune-inflammatory HCC, and C2 was defined as elevated cell proliferative and metabolic HCC.

Additionally, we compared w previous immune-related molecular subtypes, and a Sankey diagram depicted the necroptosis subtype sample contribution of HCC patients to immune subtypes. The necroptosis subtypes C1 and C2 have tight links with immune subtypes C3 and C4, respectively (**Figure S3C**). Consistent with previous research and our results, immune subtype C3 presented the most favorable prognosis at both OS and progression-free survival (PFS) levels (p < 0.05) (**Figures 5D, E**). Taken together, two necroptosis subtypes harbored important prognosis values.

### The validation of necroptosis subtypes

**Figure 6:**
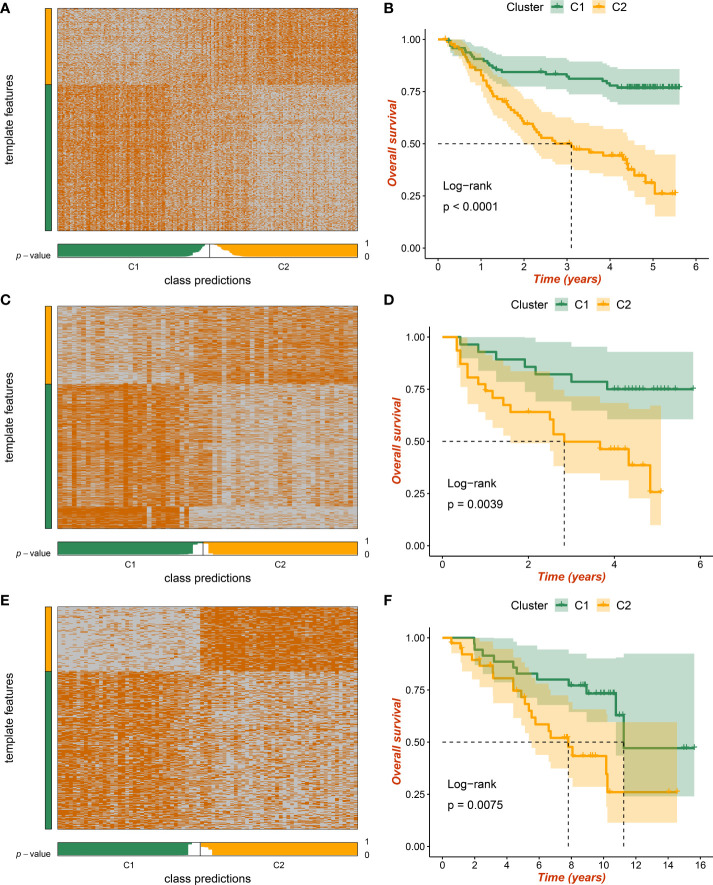
The validation of necroptosis subtypes by nearest template prediction (NTP) approach. **(A)** Validation of two heterogeneous necroptosis subtypes based on the nearest template prediction (NTP) analysis in GSE14520 dataset. **(B)** Kaplan–Meier curves of overall survival (OS) according to the necroptosis subtypes in GSE14520 dataset. **(C)** Validation of two heterogeneous necroptosis subtypes based on the NTP analysis in GSE116174 dataset. **(D)** Kaplan–Meier curves of OS according to the necroptosis subtypes in GSE116174 dataset. **(E)** Validation of two heterogeneous necroptosis subtypes based on the NTP analysis in GSE10141 dataset. **(F)** Kaplan–Meier curves of OS according to the necroptosis subtypes in GSE10141 dataset.

Three independent external datasets from distinct platforms were further retrieved to verify necroptosis subtypes, including GSE14520, GSE116174, and GSE10141 datasets. According to characteristic gene expression, the NTP approach was exploited to explore the stability and reliability of necroptosis subtypes. The characteristic genes were filtered out when soft threshold β was set as 5 using WGCNA (**Figure S3A**). Based on the most pronounced modules, the pink and red components were defined as C1- and C2-specific characteristic genes, respectively (**Figure S3B**). After prediction confidence was quantified and evaluated, samples with a false discovery rate (FDR) of less than 0.05 were extracted for subsequent investigations (**Figures 6A, C, E**). All the Kaplan–Meier analyses elaborated that C2 still presented dismal OS in GSE14520 (**Figure 6B**), GSE116174 (**Figure 6D**), and GSE10141 datasets (**Figure 6F**). Our results proved the stability and prognosis value of necroptosis subtypes *via* rigorous validation.

### The distinct molecular and clinical features

**Figure 7:**
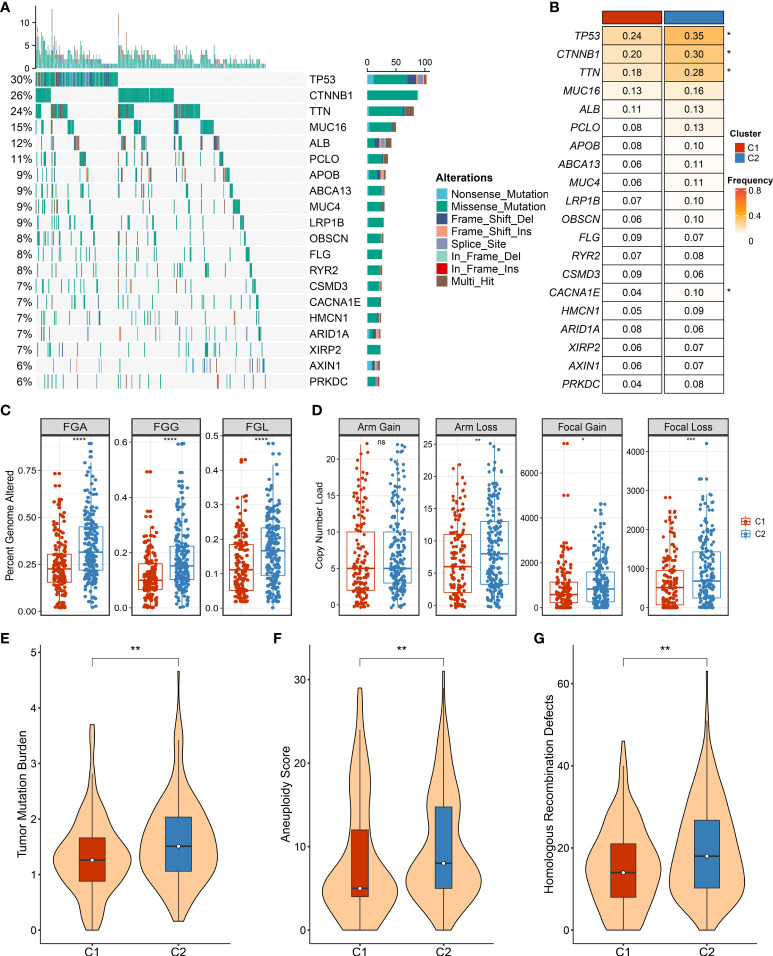
The landscape of genomic alternations in necroptosis subtypes. **(A)** The overview of somatic mutation with top 20 frequently mutated genes (FMGs) by waterfall plot. The right panel shows the mutation rate, and genes are ordered by their mutation frequencies. **(B)** Comparison of top 20 FMGs between necroptosis subtypes. **(C)** Distributions of fraction of genome alteration (FGA), fraction of genomic gained (FGG), and fraction of genome lost (FGL) between necroptosis subtypes. **(D)** Distributions of arm gain, arm loss, focal gain, and focal loss. **(E)** Distribution of tumor mutational burden (TMB) between necroptosis subtypes. **(F,G)** The comparison of aneuploidy score **(F)** and homologous recombination deficiency (HRD) **(G)** between necroptosis subtypes. ns, p >0.05, *p < 0.05, **p < 0.01, ***p < 0.001, ****p < 0.0001.

As illustrated in **Figure 7A**, the landscape of somatic mutation was explored, and the top 20 FMGs were delineated in HCC patients. Notably, C2 displayed prominently superior mutational frequency than C1 in common FMGs, especially for *TP53*, *CTNNB1*, *TNN*, and *CACNA1E* (**Figure 7B**). Although some mutations were not statistically different, there was an obvious tendency for high mutational frequency in C2, such as *PCLO* and *PRKDC* (**Figure 7B**). To further dissect the genomic variations, the CNV in bases, fragments, and chromosome arms were compared and evaluated between the distinct necroptosis subtypes (**Figures 7C, D**). Strikingly, C2 presented conspicuous CNV and harbored much more deletions at bases, fragments, and chromosome arm levels (**Figures 7C, D**). For amplification alterations, C2 also behaved with a higher burden at bases and fragments, excluding chromosome arm levels (**Figures 7C, D**). In line with somatic mutation and CNV, C2 possessed a more elevated burden including TMB, aneuploidy score, and HRD compared to C1 (**Figures 7E–G**). Taken together, C1 was regarded as a stable genome subtype, while C2 was characterized by high genomic instability.

**Figure 8:**
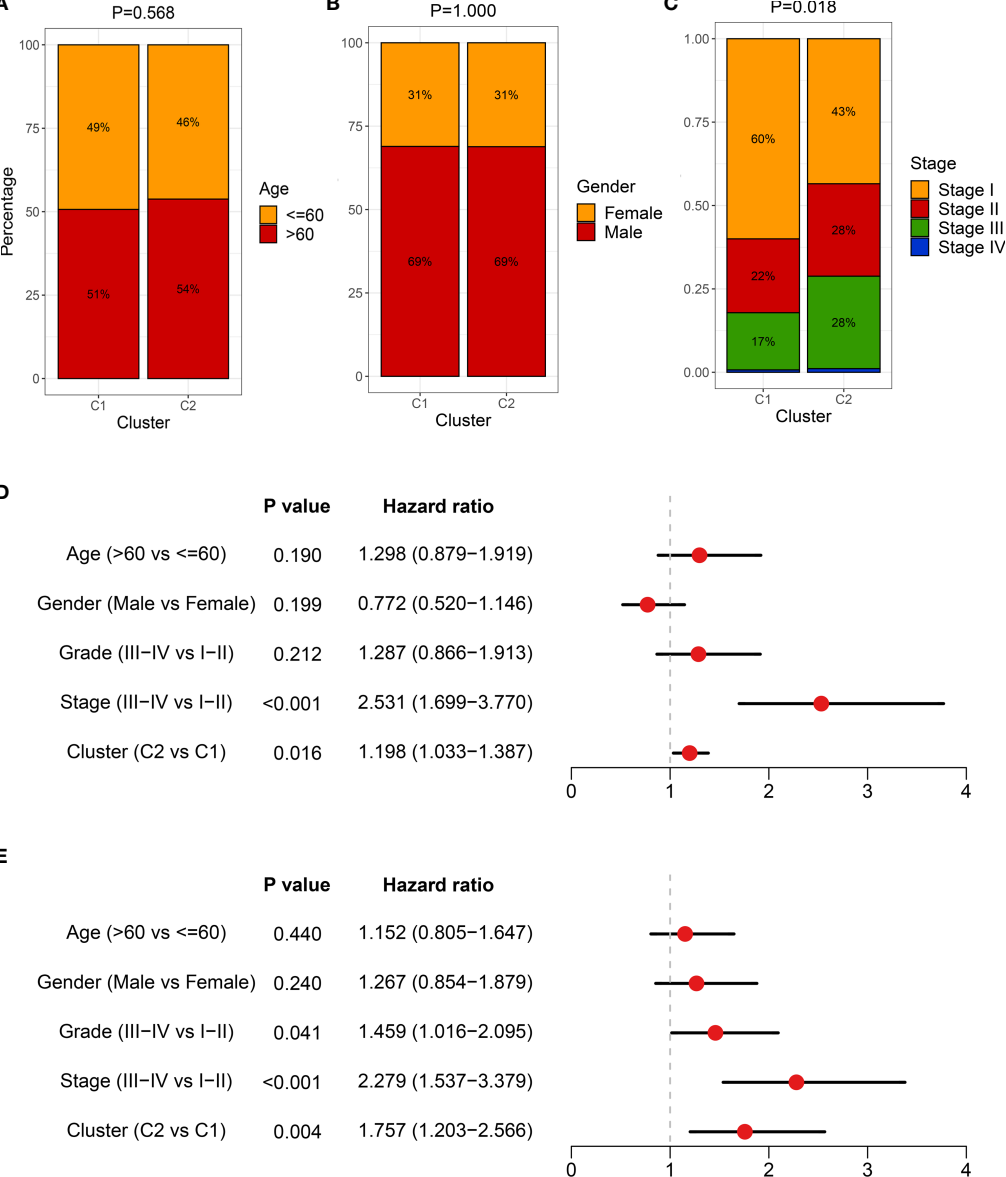
The clinical characteristics of heterogeneous necroptosis subtypes. **(A–C)** Composition percentage of clinical characteristics, including age **(A)**, gender **(B)**, and American Joint Committee on Cancer (AJCC) stage **(C)**, respectively. **(D)** Multivariate Cox regression analysis of overall survival (OS) in TCGA-LIHC dataset. **(E)** Multivariate Cox regression analysis of recurrence-free survival (RFS) in TCGA-LIHC dataset.

In addition, we also deciphered clinical features between necroptosis subtypes, encompassing age, gender, and AJCC stage (**Figures 8A–C**). There was no statistical significance between age and gender, while patients in C2 had advanced clinical stages, implying a more malignant phenotype (**Figures 8A–C**). Considering that C2 is characterized by elevated cell proliferative and metabolic activities and superior clinical stage, the poor prognosis was further explained. Combining these clinical traits, multivariate Cox regression analysis indicated that both AJCC stage and necroptosis subtypes were independent risk indicators for assessing OS (**Figure 8D**). A similar discovery also appeared in predicting RFS in TCGA-LIHC dataset (**Figure 8E**). Therefore, necroptosis subtypes could be a promising tool to evaluate the prognosis of HCC patients.

### Integrative assessment of immunotherapy for hepatocellular carcinoma patients

**Figure 9:**
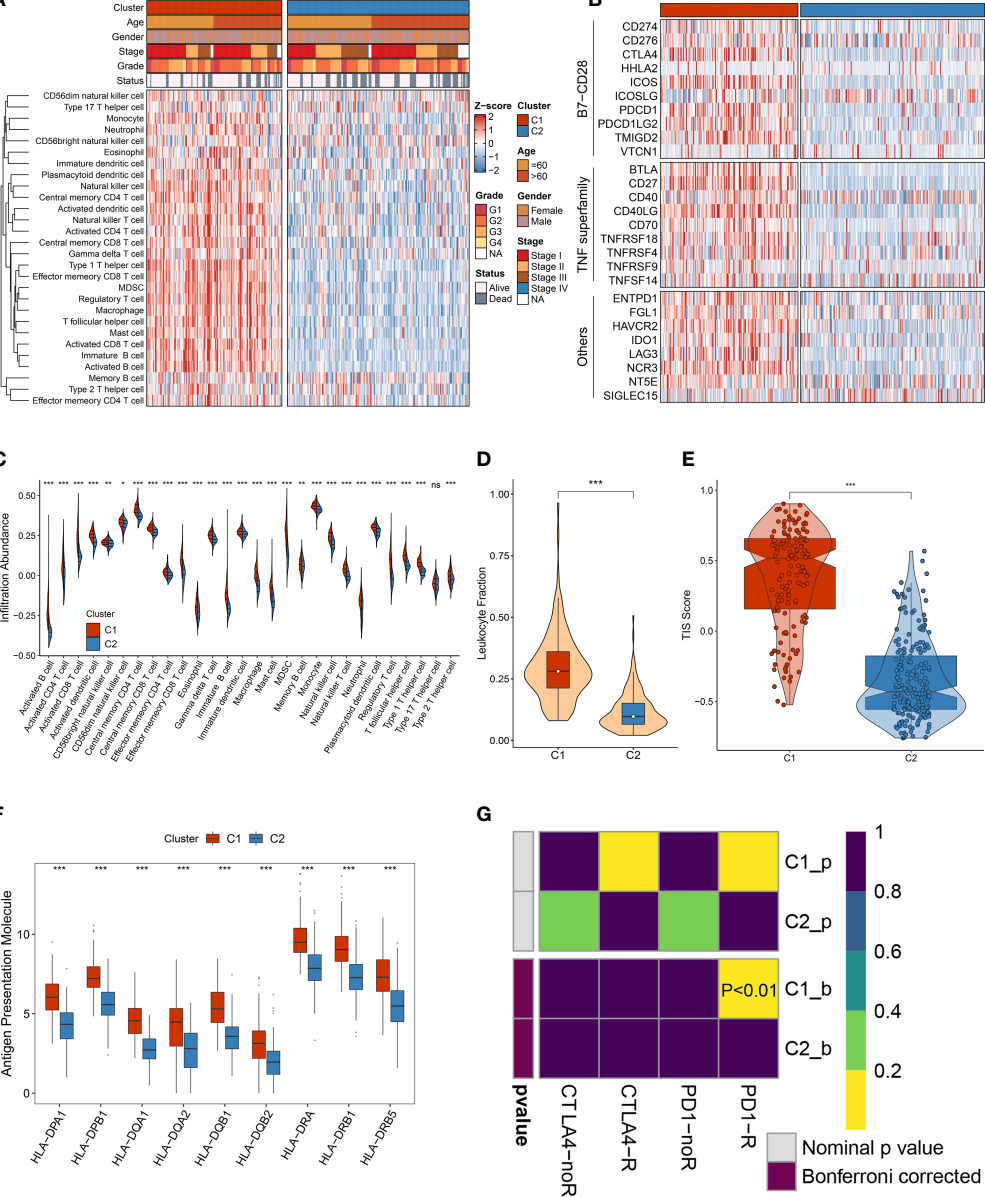
The immune landscape of heterogeneous necroptosis subtypes. **(A)** The infiltration abundance of 28 immune cell subsets was evaluated by single-sample gene set enrichment analysis (ssGSEA) algorithm. **(B)** The immune checkpoint profiles of necroptosis subtypes, including B7-CD28 superfamily, TNF superfamily, and other molecules. **(C)** Distribution of 28 immune cell infiltration between necroptosis subtypes in TCGA-LIHC dataset. **(D)** The comparison of leukocyte fraction between necroptosis subtypes. **(E)** Distribution difference of T-cell inflammatory signature (TIS) prediction scores between necroptosis subtypes. **(F)** Distribution of nine HLA molecular expressions between necroptosis subtypes. **(G)** Submap analysis exhibited that C1 could be more sensitive to the anti-PD-1 therapy (p < 0.01**)** ns, p >0.05, *p < 0.05, **p < 0.01, ***p < 0.001.

The necroptosis subtypes displayed pronounced heterogeneity in several aspects, including prognosis, genomic variations, biological characteristics, and clinical features. To determine optimal treatment and further facilitate the clinical outcome are urgently needed. To bridge this gap, immune gene sets of stored 28 immune cells were accessed from a previous study (**Table S3**). Compared to C2, C1 was inclined to the ‘immune-hot’ subtype, which harbored much more immune cell infiltration in TME (**Figure 9A**). C1 also exhibited more elevated expression of 27 ICP molecules, including the B7-CD28 superfamily, TNF superfamily, and other molecules (**Figure 9B**). Numerous immunologic effector cells were gathered in C1 subtypes, such as activated B cell, CD4+ T cell, and CD8+ T cell, indicating stronger antitumor killing ability (**Figure 9C**). In addition, co-stimulatory and co-stimulatory ICPs have a prevalently high expression in C1 relative to C2 (**Figures S4A, B**). All these immune cell infiltrations and molecule expression have promising potential for immunotherapy. Both leukocyte fraction and TIS score were higher in C1 rather than C2, implying more responsiveness to immunotherapy (**Figures 9D, E**). Owing to the elevated expression of HLA molecules, we presumed that patients in C1 possessed a better capability of delivering antigens (**Figure 9F**). Furthermore, the Submap approach was executed to identify populations of elevated response to immunotherapy, and the results also indicated that C1 populations might gain favorable clinical benefits (**Figure 9G**). Overall, all evidence highlighted that C1 had enhanced immune repertories to perform antitumor power and might be more applicable to immunotherapy.

**Figure 10:**
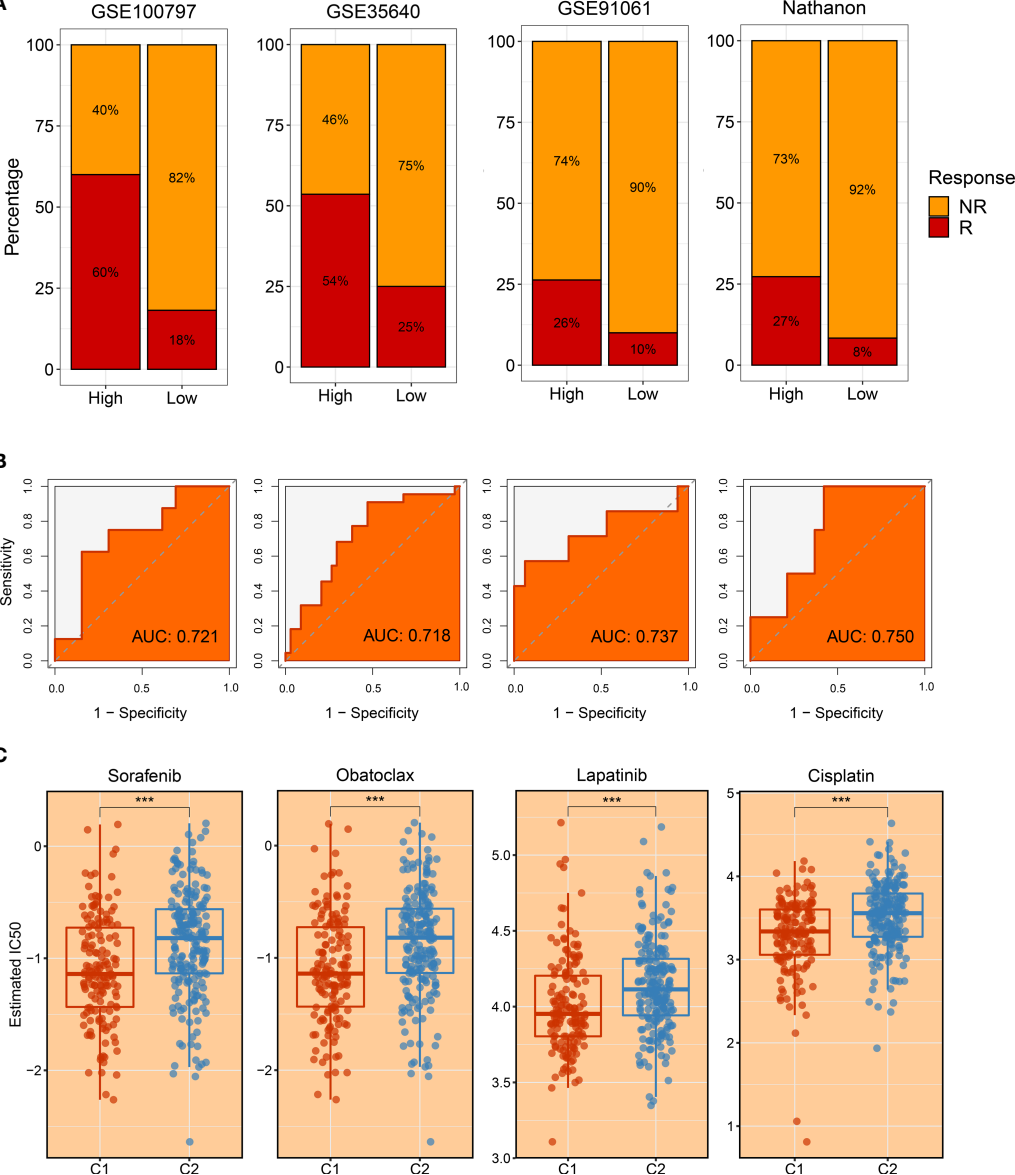
The evaluation of immunotherapy and drug-associated treatment. **(A)** The immunotherapy response ratio of risk scores measured by characteristic genes in GSE100797, GSE35640, GSE91061, and Nathnaon datasets. **(B)** Receiver operating characteristic (ROC) curves of risk scores to predict the benefits of immunotherapy in GSE100797, GSE35640, GSE91061, and Nathnaon datasets. **(C)** The promising therapeutic agents for patients in C1. ***p < 0.001.

Subsequently, four immunotherapy datasets encompassed 41 responders, and 98 non-responders were used to estimate the applicable potential of immunotherapy for C1 populations. According to characteristic genes of C1, a score was calculated for each patient using the ssGSEA method; and all patients were assigned into high and low groups by the median value. Of note, patients in the high group displayed stronger sensitivity to immunotherapy in GSE100797 (60% vs 18%), GSE35640 (54% vs 25%), GSE91061 (26% vs 10%), and Nathnaon datasets (27% vs 8%) (**Figure 10A**). The accuracy of immunotherapy evaluation was further examined by area under the curve (AUC) values in GSE100797 (0.721), GSE35640 (0.718), GSE91061 (0.737), and Nathanon datasets (0.750) (**Figure 10B**). Collectively, patients in C1 were more suitable for immunotherapy.

### Development of potential therapeutic drugs for hepatocellular carcinoma patients

**Figure 11:**
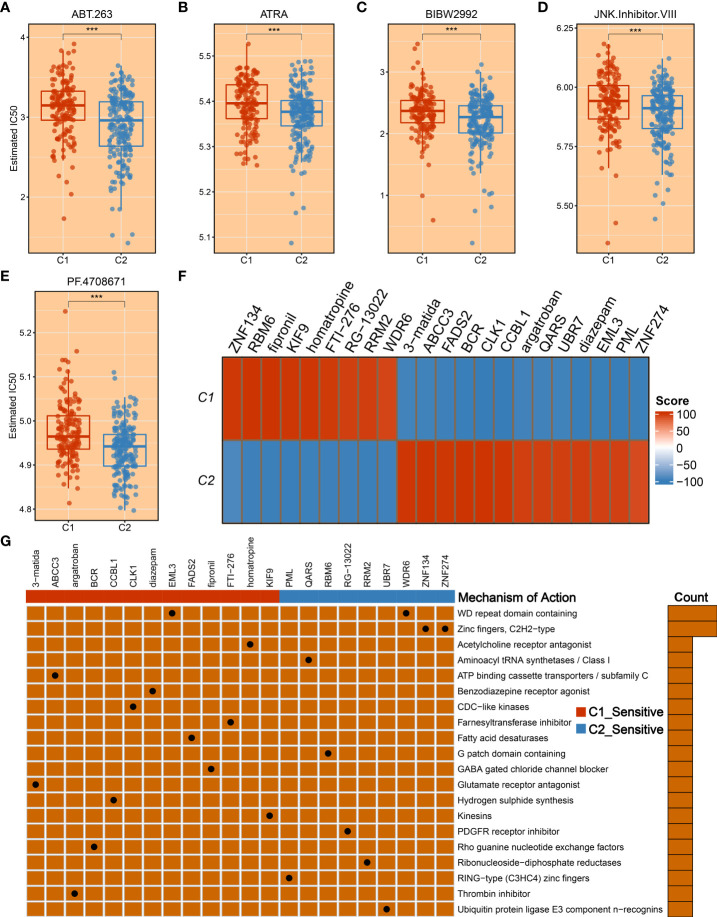
The development of promising therapeutic agents and description of targeted molecules and corresponding pathways. **(A–E)** The promising therapeutic agents for patients in C2, including ABT.263 **(A)**, ATRA **(B)**, BIBW2992 **(C)**, JNK.Inhibitor.VIII **(D)**, and PF.4708671 **(E)**. **(F)** Heatmap of enrichment score generated from potential therapeutic compounds. **(G)** The description of the mode of action (MoA) of compounds targeting corresponding molecular pathways. ***p < 0.001.

To tailor individualized clinical treatment for each patient, the ridge regression model and CMap database were employed to identify latent therapeutic drugs. One strategy used a ridge regression model based on the *pRRophetic* package; drug sensitivity data were calculated and quantified *via* half-maximal inhibitory concentration (IC50), developing candidate drugs. Our study determined that patients in C1 might harbor a superior response to sorafenib, obatoclax, lapatinib, and cisplatin because of lower IC50 values (**Figure 10C**). In addition, patients in C2 displayed more sensitivity to ABT.263, ATRA, BIBW2992, JNK.Inhibitor.VIII, and PF.4708671 (**Figures 11A–E**). All these candidate drugs might bring promising desirable efficacy for specific HCC patients. Another approach was combining the CMap database; seeking opposite expression patterns between molecular subtype and disease phenotype was performed to identify potential compounds and elucidate the mode of action (MoA). A total of 22 drugs harbored individualized therapeutic potential for necroptosis subtypes (**Figure 11F**). Moreover, the pathways of these candidate drugs were described, which could be utilized to develop more curative drugs (**Figure 11G**). Different drugs and targeted pathways might guide individualized therapy patterns to improve clinical benefits.

## Discussion

Currently, tumor heterogeneity is focused on clinical ‘decision-making’ mainly since patients display different degrees of treatment response and distinct clinical outcomes. Exploring the tumor heterogeneity is conducive to increasing the knowledge of HCC and seeking more appropriate treatment strategies (29, 30). This study comprehensively elaborated on the relationships between inter-tumoral heterogeneity and necroptosis. Based on previously published literature, a total of 115 necroptosis-associated genes were enrolled. Subsequently, using the WGCNA algorithm and univariate Cox regression analysis, NALRs were identified, which were further employed to decode heterogeneous necroptosis subtypes according to the consensus clustering approach. With the aid of various evaluation indicators, such as consensus score, CDF curve, and PAC, two necroptosis subtypes were determined. Moreover, the NTP approach elucidated the robustness and reproducibility of necroptosis subtypes. Further clinical and prognostic analysis indicated that C1 presented a favorable prognosis while C2 displayed a poor prognosis and superior AJCC stage, and necroptosis subtypes could serve as an independent prognostic indicator. All these discoveries delineated that necroptosis might play a key role in HCC tumorigenesis and increased interest in more in-depth explorations.

The heterogeneous clinical outcome is generally mirrored by distinct molecular characteristics, such as genomic alterations (31, 32). Two necroptosis subtypes displayed distinct genomic variations in multi-omics levels. Through systematic investigations on genomic variants burden, C1 was prone to genomic stability, while C2 referred to genomic instability owing to conspicuous genomic alterations including TMB, FGA, FGG, and FGL and alterations at chromosome arm and focal levels. The highly genomic instability is reported to be linked with immune evasion, enriched proliferative phenotype, and worse prognosis (32). The pronounced somatic mutations were also observed in patients in C2, including *TP53*, *CTNNB1*, *TNN*, and *CACNA1E*. Previous studies had revealed that the mutation of *TP53* and *CTNNB1* mediated cell cycle and WNT signaling pathways, resulting in tumor progression (33). In addition, underlying biological mechanisms regulate cancer biological behaviors, which harbor intense association with prognosis (34). With the use of enrichment analysis, the high genomic instability phenotype C2 also possessed numerous proliferation and metabolism-related pathways, further favoring malignant features. The favorable prognosis phenotype C1 showed more dynamic immune-inflammatory pathways, implying the superior potential for immunotherapy response. Based on previous immune subtypes (23), we observed a higher proportion of inflammatory C3 in the C1 subtype and lymphocyte-depleted C4 in the C2 subtype. Therefore, all the above findings suggested that C1 harbored more promising potential to perform desirable benefits for immunotherapy as compared to C2.

To further seek the optimal treatment option, we estimated the immune landscape of each patient, encompassing immune cell infiltrations, expression of diverse ICPs, distribution of HLA molecules, fraction of leukocyte cells, etc. In this study, C1 was inclined to the ‘immune-hot’ subtype as harboring abundant enrichment of numerous immune cells and various immune-related molecules. Moreover, HLA molecules are widely reported to strengthen antitumor ability by antigen presentation (35), and leukocyte fraction was conducive to enhancing cytolytic activity (36). Elevated expression of HLA molecules and a high fraction of leukocyte cells in the C1 subtype might arise in better immunotherapeutic response and more curative benefits. The CD8+ T cells are the main force and direct effectors to fight against tumors (37, 38). The CD4+ T cells work as helpers to activate CD8+ T cells, enhancing antitumor performance (39). Some classical ICPs, *CD274* and *CTLA-4*, are immunotherapeutic targets, and the higher expression implies more curative potential (40, 41). Subsequently, two popular approaches, TIS and Submap, were exploited to evaluate the immunotherapeutic efficacy of two necroptosis subtypes. Consistently, C1 still had thrilling potential benefits for immunotherapy relative to the C2 subtype. To yield more insights on immunotherapy, our study enrolled three immunotherapy datasets that contained both expression profiles and immunotherapeutic clinical information, which further provided immunotherapy recommendations for specific populations. Overall, our study throws light on precision medicine, and patients in C1 were encouraged to undergo immunotherapy.

As described above, C2 possessed significant malignant features, including worse prognosis, elevated proliferative and metabolic activities, advanced AJCC stage, and highly genomic instability. To fill this gap, we took more consideration to improving prognosis and facilitating desirable efficacy for the C2 subtype. Another matter that also needs to be considered was that a subset of patients displayed sensitivity to specific drugs, while some of them are suffering from side effects (42). Therefore, we developed a ridge regression model to identify potential therapeutic drugs for HCC patients. Based on expression profiles and large-scale drug sensitivity data, five potential therapeutic drugs were developed for C2, including ABT.263, ATRA, BIBW2992, and JNK.Inhibitor.VIII, and PF.4708671. Among these candidate drugs, ABT.263 is one Bcl-2 protein family inhibitor, which could strengthen autophagy to suppress tumor growth by enhancing *LC3-II* and inhibiting *p62* gene expression (43). The ATRA is a differentiation inducer of tumor-initiating cells and plays an antitumor role in tumorigenesis by anti-proliferative and pro-apoptotic behaviors (44). The PF.4708671 is one S6K1-specific inhibitor, which impacts pro-apoptotic function by blocking the mTORC1-S6K1 signaling (45). The BIBW2992, also known as afatinib, is a second-generation tyrosine kinase inhibitor (TKI) that could target *EGFR* and *HER2* molecules, resulting in tumor suppression (46). Moreover, we delineated other representative therapeutic drugs and latent mechanisms of action using Camp datasets. Given the above, these potential therapeutic drugs may provide novel hope for HCC treatment and deliver precision medicine.

To improve clinical outcomes and provide treatment recommendations, two necroptosis subtypes were ultimately identified in this study. Although the strengths of our study are promising, some limitations should be acknowledged. a) All patients retrieved in the study were from retrospective studies, so the conclusions need to be validated by multi-center prospective studies. b) Owing to multi-omics data being deficient in validation datasets, more differences in genomic alterations should be depicted in the future. c) Much more clinical trial research is needed for further delineation and exploration, and more eligible patients with treatment information need to be enrolled in further studies.

In conclusion, this study uncovered the tumor heterogeneity and provided two necroptosis subtypes in HCC. The heterogeneous molecular characteristics were further revealed in necroptosis subtypes, which harbored distinct clinical outcomes, genomic landscape, biological behaviors, and immune characteristics. Patients in C1 were more prone to obtain conspicuous efficacy from immunotherapy. For patients in C2, our study developed potential therapeutic drugs that had desirable efficacy for patients, improving their prognosis. Overall, this work afforded new insights into tumor heterogeneity based on necroptosis and tailored individualized treatment strategies for HCC patients.

## Data availability statement

The original contributions presented in the study are included in the article/**Supplementary material**. Further inquiries can be directed to the corresponding author.

## Author contributions

BH and SZ designed this work. BH, JG, JS, FZ, and SZ integrated and analyzed the data. BH, CS, ZW, PW, and WG wrote this manuscript. BH, JG, WG, and SZ edited and revised the manuscript. All authors approved this manuscript.

## Funding

This study was supported by the Youth Project of the National Natural Science Foundation of China (82103282) and Hepatobiliary Foundation of Henan Charity General Federation (GDXZ2019006).

## Conflict of interest

The authors declare that the research was conducted in the absence of any commercial or financial relationships that could be construed as a potential conflict of interest.

## Publisher’s note

All claims expressed in this article are solely those of the authors and do not necessarily represent those of their affiliated organizations, or those of the publisher, the editors and the reviewers. Any product that may be evaluated in this article, or claim that may be made by its manufacturer, is not guaranteed or endorsed by the publisher.
