# Supplementary material for: Necroptosis throws novel insights on patient classification and treatment strategies for hepatocellular carcinoma
Source: Front Immunol. 2022 Jul 27;13:970117. doi: 10.3389/fimmu.2022.970117 (PMC9363630; doi:10.3389/fimmu.2022.970117)
Supplement: Supplementary file 1 [file DataSheet_1.docx]

**Supplementary Figures**

- Figure S1
- Figure S2
- Figure S3
- Figure S4


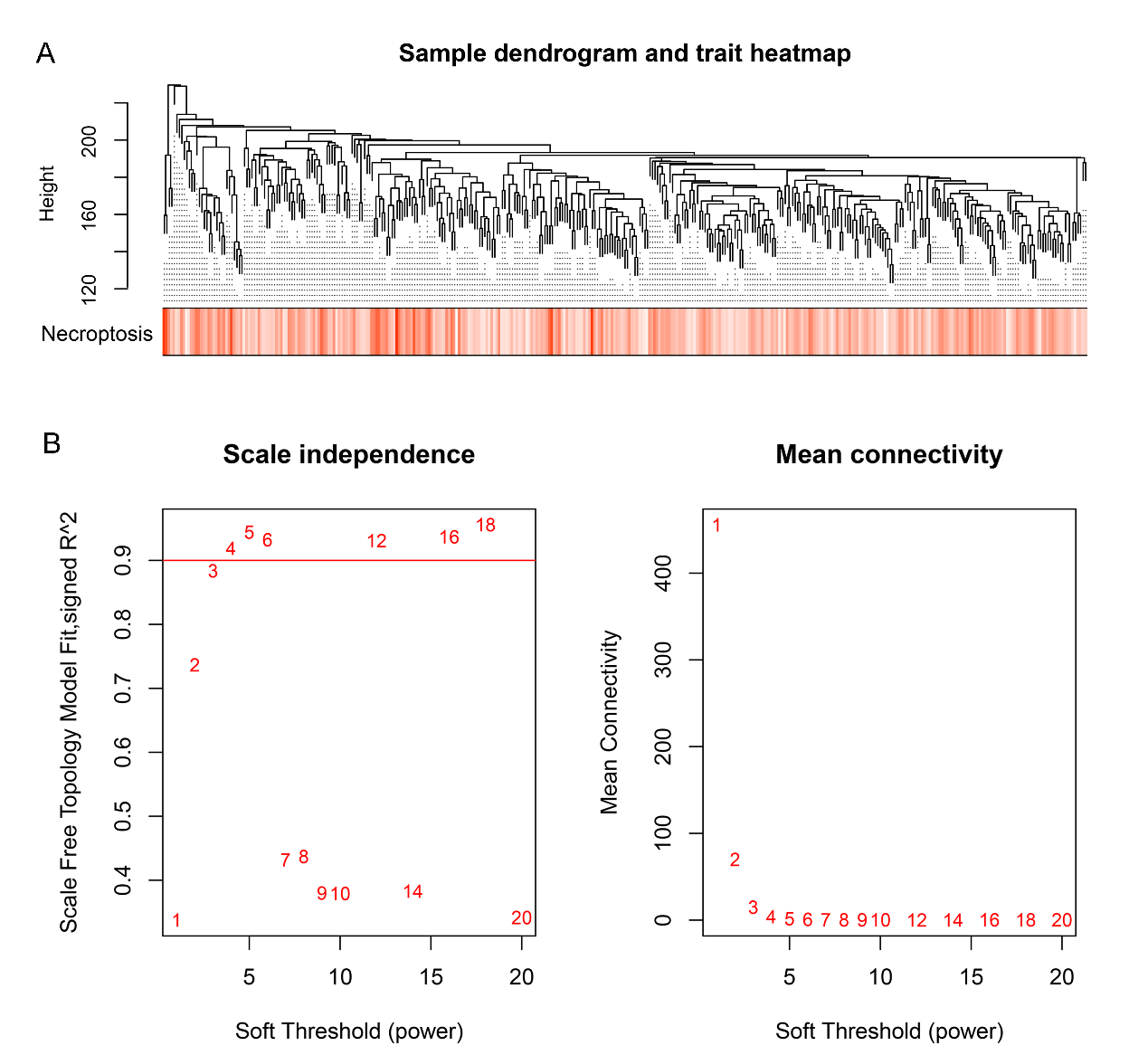


**Figure S1. The selection of soft threshold through weighted gene co-expression network analysis (WGCNA). (A).** Hierarchical clustering of the remaining samples after remove outlier HCC samples. **(B).** Analysis of network topology for different soft-threshold power by WGCNA. The left panel shows the impact of soft threshold power on the scale-free topology fit index; the right panel displays the impact of soft-threshold power on the mean connectivity.


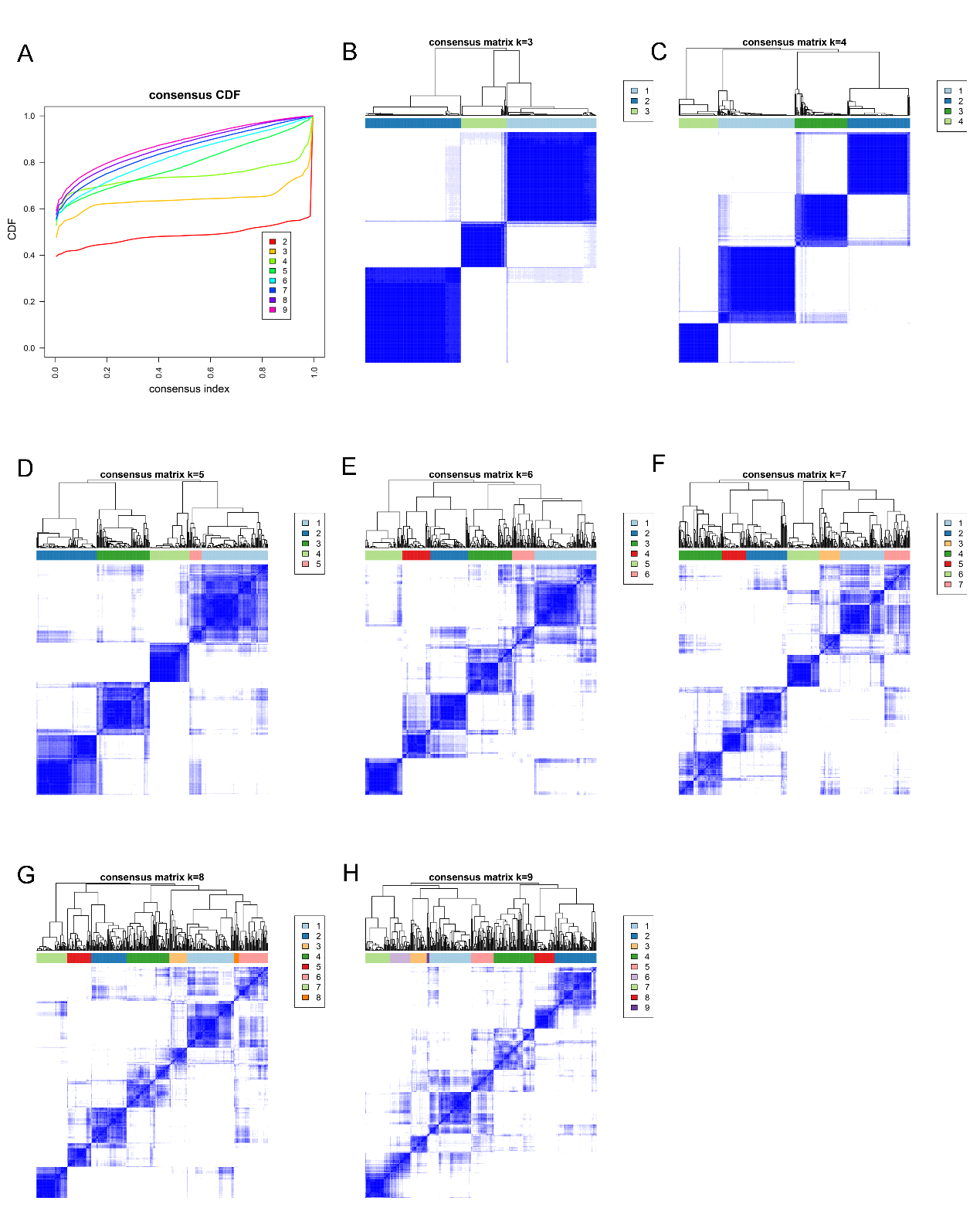


**Figure S2. The optimal rank of consensus clustering analysis.** **(A).** Consensus CDF shows a real random variable of its probability distribution based on consensus scores for different subtype numbers (k =2-9). **(B-H).** The consensus score matrix of necroptosis subtypes using consensus unsupervised clustering when various ranks (k =3-9).


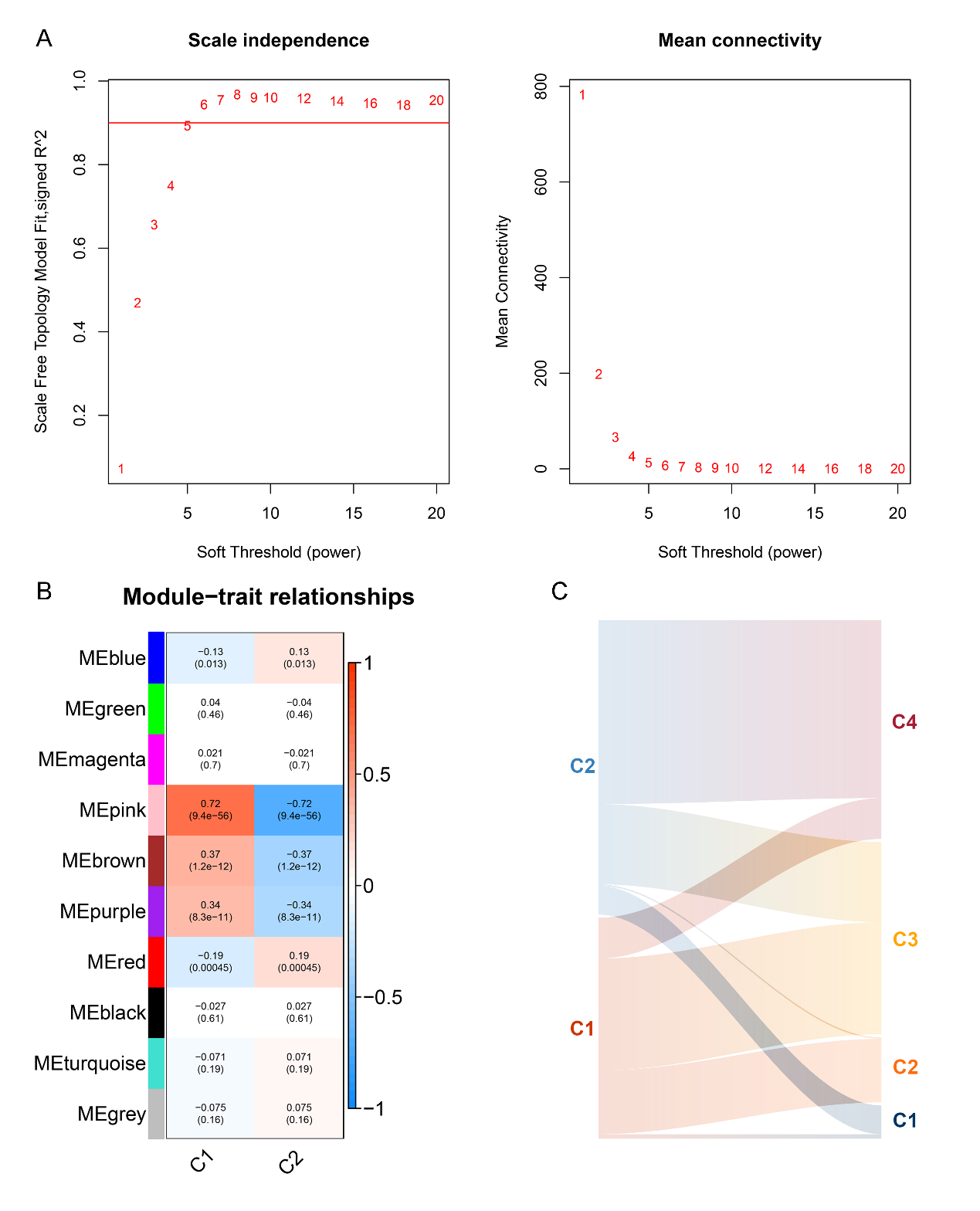


**Figure S3. Identification of characteristic genes in heterogeneous necroptosis subtypes.** **(A).** The selection of soft threshold power according to scale independence and mean connectivity. **(B).** Correlation analysis between gene modules and necroptosis subtypes. **(C).** The potential links between necroptosis subtypes and previous immune subtypes.


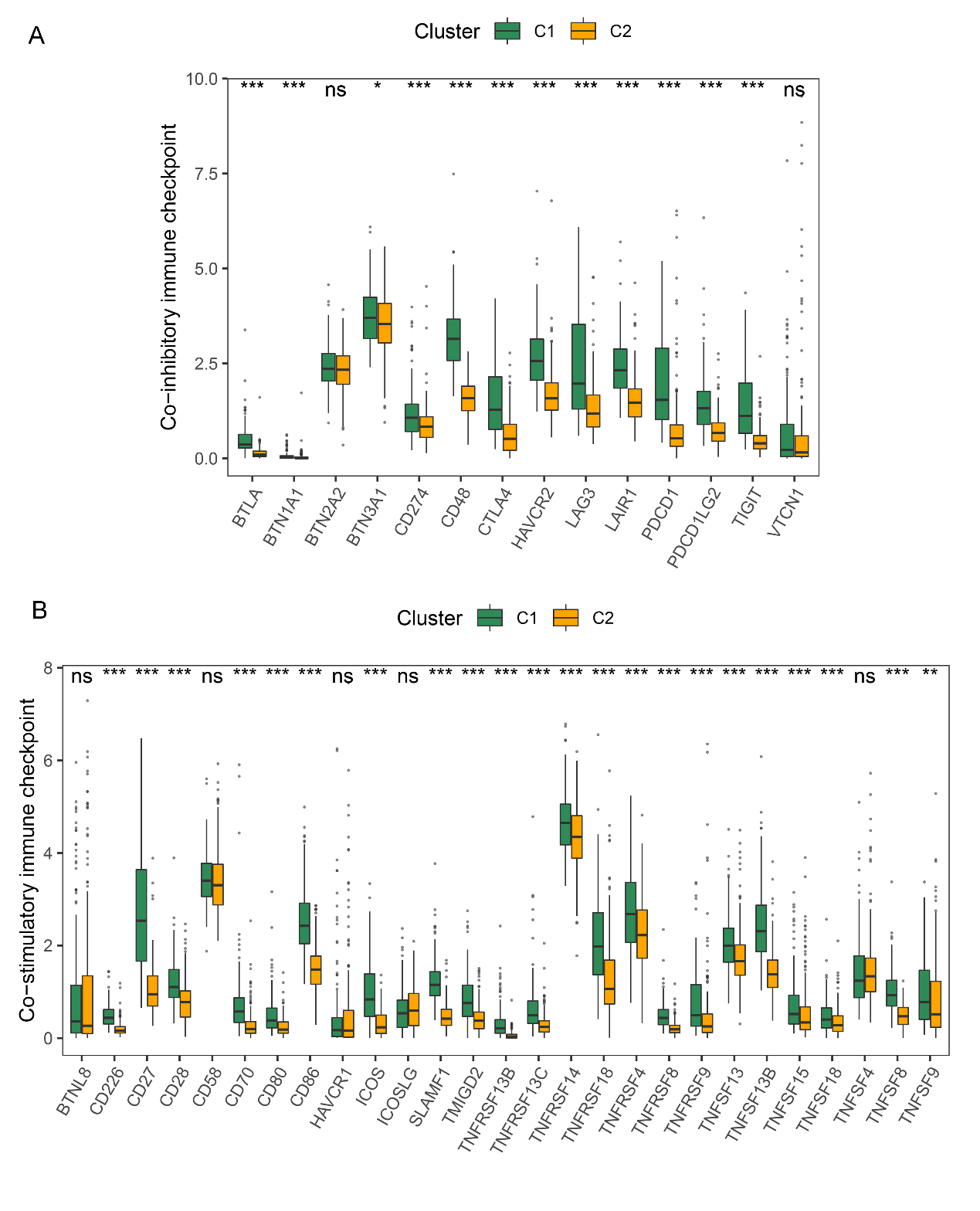


**Figure S4. The expression of co-stimulatory and co-inhibitory molecules. (A).** Distribution of co-stimulatory molecules between distinct necroptosis subtypes C1 and C2. **(B).** Distribution of co-inhibitory molecules between distinct necroptosis subtypes C1 and C2.
